# Radiogenomic Landscape of Metastatic Endocrine-Positive Breast Cancer Resistant to Aromatase Inhibitors

**DOI:** 10.3390/cancers17050808

**Published:** 2025-02-26

**Authors:** Richard Khanyile, Talent Chipiti, Rodney Hull, Zodwa Dlamini

**Affiliations:** 1 SAMRC Precision Oncology Research Unit (PORU), DSI/NRF SARChI Chair in Precision Oncology and Cancer Prevention (POCP), Pan African Cancer Research Institute (PACRI), University of Pretoria, Hatfield 0028, South Africa; richard.khanyile@up.ac.za (R.K.); talent.chipiti@up.ac.za (T.C.); rodney.hull@up.ac.za (R.H.); 2 Department of Medical Oncology, Steve Biko Academic Hospital and University of Pretoria, Pretoria 0001, South Africa

**Keywords:** breast cancer, endocrine-positive, aromatase inhibitors, resistance mechanisms, genetic and epigenetic alterations, radiogenomics, imaging techniques, genomic profiling, metastasis patterns, personalized treatment

## Abstract

Breast cancer poses a significant global health challenge and includes various subtypes, such as endocrine-positive, HER2-positive, and triple-negative. Endocrine-positive breast cancer, characterized by estrogen and progesterone receptors, is commonly treated with aromatase inhibitors. However, resistance to these inhibitors can hinder patient outcomes due to genetic and epigenetic alterations, mutations in the estrogen receptor 1 gene, and changes in signaling pathways. Radiogenomics combines imaging techniques like MRI and CT scans with genomic profiling methods to identify radiographic biomarkers associated with resistance. This approach enhances our understanding of resistance mechanisms and metastasis patterns, linking them to specific genomic profiles and common metastasis sites like the bone and brain. By integrating radiogenomic data, personalized treatment strategies can be developed, improving predictive and prognostic capabilities. Advancements in imaging and genomic technologies offer promising avenues for enhancing radiogenomic research. A thorough understanding of resistance mechanisms is crucial for developing effective treatment strategies, making radiogenomics a valuable integrative approach in personalized medicine that aims to improve clinical outcomes for patients with metastatic endocrine-positive breast cancer.

## Simple Summary

Breast cancer is a serious health issue worldwide. The endocrine-positive type, which grows in response to estrogen and progesterone, is the most common. Treatments often include aromatase inhibitors. However, some patients become resistant to treatment through genetic changes or changes in cellular pathways. Radiogenomics is a new method that combines imaging processes like MRIs and CT scans with genetic studies. This approach facilitates our understanding of how cancer resists treatment and spreads, especially to the bones or brain. This method helps create personalized treatment plans by connecting imaging results with genetic profiles. With the current state of technology, radiogenomics is looked at as being able to improve the diagnosis, treatment, and outcomes of patients with hard-to-treat endocrine-positive breast cancers.

## 1. Introduction

Breast cancer (BC) is one of the most common cancers worldwide, significantly affecting global health, as it contributes to a large portion of cancer-related illnesses and deaths. In women, it is the most prevalent malignant tumor and poses a serious threat to the global female population. The 2020 global BC statistics report projected 2.261 million new cases and 685,000 deaths worldwide, solidifying BC as the leading malignant tumor globally. Although some cancer cases are unavoidable, governments can implement health interventions to minimize exposure to known risk factors, including environmental, lifestyle, dietary, and metabolic influences. Gaining insights into the roles of modifiable risk factors in the global burden of breast cancer (BC) is essential for developing effective cancer control strategies at both local and global levels [1].

Breast cancer is a highly diverse disease with different subtypes. These subtypes are typically classified into four groups based on the expression of hormone receptors detected using immunohistochemistry. The main types of breast cancer include estrogen receptor-positive (ER+), progesterone receptor-positive (PR+), and human epidermal growth factor receptor-positive (HER2+). In contrast, triple-negative breast cancer (TNBC) is unique because it does not express any of these receptors [2].

Among these, endocrine-positive breast cancer, characterized by the expression of estrogen receptors (ER) and progesterone receptors (PR), represents the most common subtype, accounting for approximately 70–80% of all breast cancer cases [3]. This subtype is generally associated with a better prognosis than HER2-positive and TNBC, with a more indolent disease course and favorable response to hormone-based therapies. Increased expression of PR is linked to better overall survival and longer times to recurrence and treatment failure or progression. Conversely, lower levels are generally associated with a more aggressive disease course, rapid and frequent recurrence, and a poorer prognosis [4].

**Figure 1:**
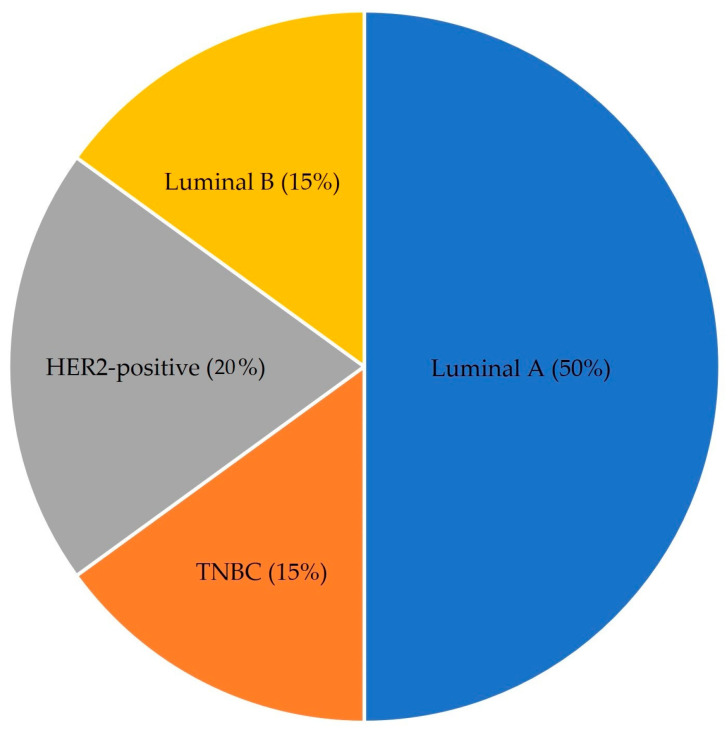
Chart showing the proportion of breast cancer subtypes. This pie chart represents the distribution of breast cancer subtypes, indicating the relative frequency of each subtype, with Luminal A being the most prevalent with a frequency of 50%. This is followed by HER2-positive with a frequency of 20%, Luminal B, and TNBC. This distribution is useful for understanding the variations in breast cancer and guiding treatment approaches based on subtype characteristics. Luminal B and TNBC had the lowest frequencies of 15%.

The chart in Figure 1 shows the frequency of the four subtypes of breast cancer. Luminal A (50%) is the most common subtype, accounting for half of all cases. Luminal A tumors typically have a favorable prognosis and are often hormone receptor-positive, making them responsive to hormonal therapy. The HER2-positive subtype represents 20% of cases and is characterized by overexpression of the HER2 protein. HER2-positive breast cancers are often more aggressive but may respond well to targeted therapies like Herceptin. Luminal B accounts for 15% of cases. Similar to Luminal A, it is usually hormone receptor-positive but has a higher proliferation rate and may require additional treatments like chemotherapy. Chemotherapy agents like anthracyclines (e.g., doxorubicin) and taxanes (e.g., paclitaxel) are effective in treating the Luminal B breast cancer subtype due to its higher proliferative index. These agents target rapidly dividing cells and, when combined with endocrine therapy, significantly improve the survival outcomes of hormone receptor-positive Luminal B patients [5]. Triple-negative breast cancer (TNBC) also comprises 15% of cases and lacks hormone receptors and HER2, making it harder to treat with targeted therapies. It is often managed with chemotherapy in conjunction with surgery. Research on breast cancer subtypes indicates that distinct molecular profiles significantly influence treatment strategies and prognostic outcomes. The Luminal A and B subtypes, which express hormone receptors, often respond favorably to hormonal therapies. However, the treatment plans must be tailored to specific receptor statuses and proliferation rates.

The molecular characterization of the Luminal A subtype shows that apart fom expressing ER and PR, it is also characterized by low Ki-67 levels, contributing to a favorable prognosis and responsiveness to hormonal therapies [2,6]. In contrast, Luminal B shows higher Ki-67 levels and may have *BRCA2* mutations, which may explain how it responds to hormonal or chemotherapy, and its moderate prognosis [4]. HER2-positive cases often exhibit high Ki-67 expression, a variable ER/PR presence, and HER2 amplification, with treatment options including hormonal therapy, chemotherapy, and Herceptin [2]. Finally, while TNBC lacks ER, PR, and HER2, this subtype also frequently involves mutations in *p53* and *BRCA1*, which appear to be important factors contributing to its poor prognosis. The treatment of TNBC relies on chemotherapy and experimental treatments due to limited targeted therapy options [3].

**Figure 2:**
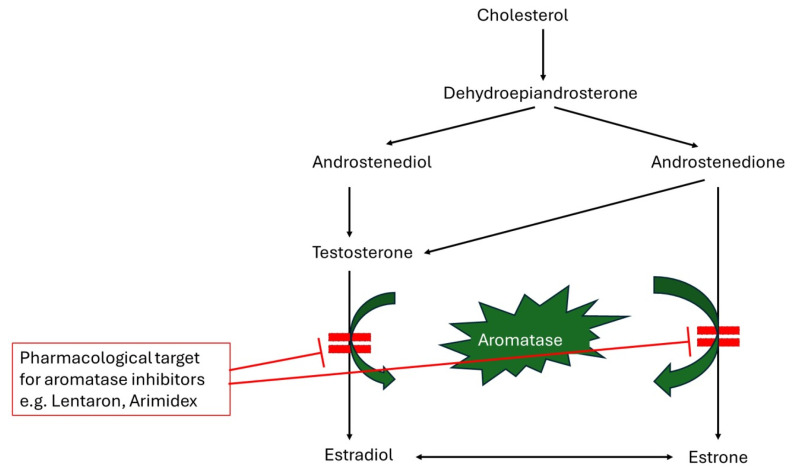
Biosynthetic pathways of steroid hormones and aromatase inhibition. This figure illustrates the biosynthetic pathways for steroid hormones, beginning with cholesterol as the precursor. It shows the enzymatic steps leading to the production of various steroid hormones, including aldosterone, cortisol, estrone, and estradiol.

The human aromatase enzyme is vital for estrogen biosynthesis, catalyzing the final and rate-limiting step that converts androgens, such as testosterone and androstenedione, into estrogens like estradiol and estrone. This conversion occurs through a series of oxidation reactions and primarily takes place in the ovaries, adrenal glands, and fat tissues. Aromatase plays a key role in regulating estrogen levels, which are important for reproductive health, bone density, and cardiovascular function. Additionally, it is a significant target in cancer therapies for hormone-sensitive cancers, such as breast cancer, where elevated estrogen levels can stimulate tumor growth. Its high expression in breast cancer tissues leads to elevated estrogen levels, making aromatases crucial targets in breast cancer treatment. Although present in various tissues, including the ovary, bone, adipose tissue, and brain, aromatase expression outside the gonads and brain is uniquely regulated in primates by tissue-specific promoters. As the only enzyme capable of aromatizing a six-membered ring, aromatase serves as the body’s sole source of estrogen [7]. Figure 2 highlights the role of the enzyme aromatase in converting androstenedione and testosterone into estrone and estradiol, respectively. The figure shows the enzymatic conversion of cholesterol leading to the production of key hormones like estrone and estradiol. This pathway is critical for understanding how steroid hormones are synthesized and where pharmacological interventions can inhibit estrogen production, particularly in the context of hormone-dependent cancers like breast cancer.

**Figure 3:**
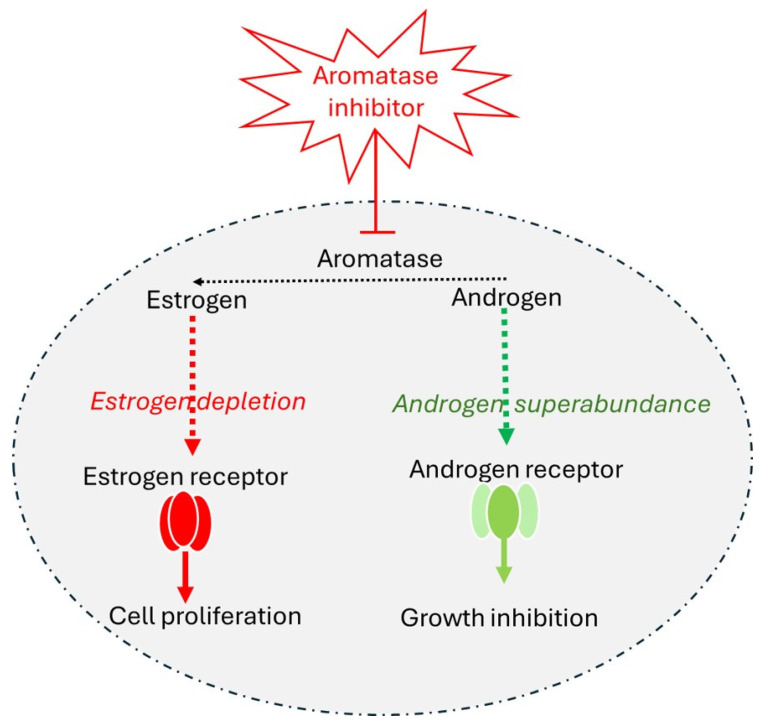
The mechanism of action of aromatase inhibitors. This figure illustrates the mechanism by which aromatase inhibitors reduce cell proliferation in estrogen receptor-positive cells. Aromatase is an enzyme that converts androgen to estrogen, supporting cell proliferation through estrogen receptor activation. Aromatase inhibitors block this enzyme, leading to estrogen depletion (red path) and thereby reducing estrogen receptor activation and downstream cell proliferation [12]. This review examines the radiogenomic landscape of metastatic, endocrine-positive breast cancer resistant to aromatase inhibitors. It discusses the connections between imaging phenotypes and molecular changes driving resistance, as well as emerging biomarkers and innovative therapeutic strategies. Understanding these mechanisms is crucial for enhancing disease management and improving patient outcomes.

Due to aromatase’s central role in estrogen production, aromatase inhibitors have become a cornerstone in the treatment of endocrine-positive breast cancer, particularly in postmenopausal women. By inhibiting the aromatase enzyme and thus blocking the conversion of androgens to estrogen, aromatase inhibitors reduce estrogen levels [8]. Figure 3, illustrates how blocking aromatase with inhibitors (e.g., Lentaron and Arimidex) leads to estrogen depletion and androgen superabundance. This reduction in estrogen levels decreases the activation of estrogen receptors, curbing cell proliferation, and limiting the growth of estrogen receptor (ER)-positive cancer cells, while the increase in androgens promotes the activation of androgen receptors, enhancing growth inhibition.

Aromatase inhibitors are widely used as adjuvant therapy and have demonstrated substantial efficacy in lowering the recurrence risk and improving the survival rates in patients with early-stage and metastatic breast cancer. Initially, patients often respond well to aromatase inhibitor therapy, experiencing significant clinical benefits.

Aromatase inhibitors are classified into two main types: Type 1 steroidal inhibitors and Type 2 non-steroidal inhibitors. Early nonspecific inhibitors, such as aminoglutethimide, were developed first, followed by selective inhibitors that are no longer in clinical use, including formestane (Type 1), fadrozole, rogletimide, and vorozole (Type 2). Currently, the selective oral aromatase inhibitors used in clinical practice include the steroidal inhibitor exemestane (Aromasin^®^, Pfizer Medical Information, New York, NY, USA), the non-steroidal inhibitors anastrozole (Arimidex^®^, AstraZeneca, Cambridge, UK) and letrozole (Femara^®^, Novartis Pharmaceuticals Corporation, Basel, Switzerland) [7].

Aromatase inhibitors play a critical role in managing hormone receptor-positive breast cancers, particularly in postmenopausal women with estrogen receptor-positive tumors. By reducing estrogen levels, aromatase inhibitors effectively control cancer growth and have demonstrated significant efficacy in lowering recurrence rates and improving survival, making them a key component of standard adjuvant therapy [8]. This targeted approach underscores the importance of subtype-specific therapies in enhancing outcomes for breast cancer patients.

However, the problem of resistance to aromatase inhibitors has emerged as a critical challenge in the management of endocrine-positive breast cancer. Resistance, which can be either de novo (present at the outset) or acquired after initial responsiveness, is clinically significant as it leads to disease progression despite ongoing therapy [9]. The prevalence of resistance is concerning, with estimates suggesting that nearly 30–40% of patients treated with aromatase inhibitors will eventually develop resistance, leading to poor outcomes and limited treatment options [10,11]. This resistance is associated with a more aggressive disease course, increased metastasis, and reduced overall survival, highlighting the urgent need for novel therapeutic strategies and a deeper understanding of the radiogenomic landscape underlying this phenomenon. Recent advances in radiogenomics, which integrate imaging data with genomic information, have begun to shed light on the molecular mechanisms driving aromatase inhibitor resistance, offering new avenues for personalized treatment approaches [12,13]. Addressing aromatase inhibitor resistance in metastatic endocrine-positive breast cancer is essential for improving patient outcomes and represents a significant area of ongoing research.

## 2. Breast Cancer Prevalence

Breast cancer represents a significant global health challenge, and is the most prevalent cancer among women. In 2020, approximately 2.3 million new cases were reported, accounting for 24.5% of all female cancer diagnoses and resulting in around 685,000 deaths worldwide [14]. The incidence of breast cancer exhibits considerable regional disparities, with higher rates observed in high-income countries. For instance, the United States has seen an annual increase of about 0.5% in breast cancer cases, with projections estimating 310,000 new invasive cases for 2024, including 15% among women under 50 years of age [15,16]. Conversely, low- and middle-income countries (LMICs) face significantly lower five-year survival rates, with figures as low as 66% in India and 40% in South Africa, underscoring the urgent need for improved healthcare access and early detection strategies [15]. The World Health Organization (WHO) has initiated the Global Breast Cancer Initiative, aiming to reduce mortality rates by 2.5% annually. This initiative emphasizes the importance of health promotion and a timely diagnosis to address disparities in breast cancer outcomes [14]. However, the COVID-19 pandemic has further complicated breast cancer management, leading to delays in screening and treatment, which may have exacerbated the already high rates of advanced-stage diagnoses. Studies have shown that interruptions in screening programs during the pandemic have resulted in increased diagnoses of later-stage breast cancer [17,18]. For example, a study in Brazil indicated that a two-month interruption in mammographic screening led to a 10.3% increase in stage III breast cancer cases [17].

**Figure 4:**
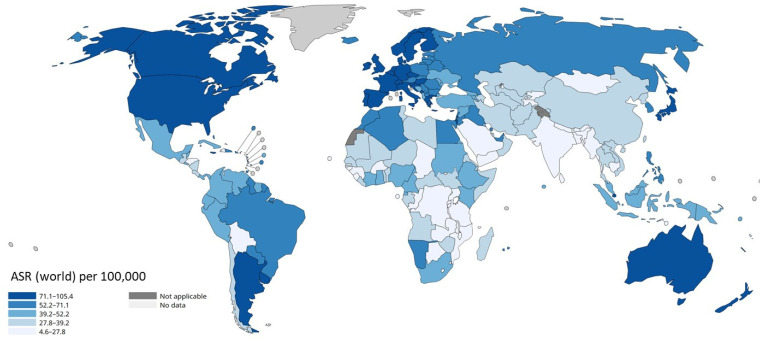
Global age-standardized breast cancer incidence rate per 100,000 in both sexes in 2022. This map shows the global incidence rates of BC per 100,000 people, standardized to the world population. The darker shades represent higher incidence rates, while lighter shades indicate lower rates. The data are sourced from The Global Cancer Observatory (GCO) (2024) [19,20].

Figure 4 illustrates the global incidence rates of BC in 2022, highlighting disparities across regions and emphasizing the need for targeted prevention measures.

The psychological impact of the pandemic on breast cancer patients has also been significant, with increased levels of anxiety and isolation reported among those undergoing treatment during this period [21,22]. Addressing these inequalities through enhanced awareness, screening, and treatment options is crucial for improving outcomes in diverse populations. The WHO’s initiative is vital in promoting early detection and improving healthcare access, particularly in LMICs where resources are limited [15]. Furthermore, the pandemic has highlighted the need for adaptive strategies in cancer care to mitigate the impact of future public health emergencies on cancer management [23,24]. As healthcare systems recover from the pandemic, prioritizing breast cancer care and ensuring equitable access to screening and treatment are essential in reducing the burden of this disease globally [25]. The World Health Organization (WHO) reports that the incidence of breast cancer has been rising globally, with an estimated 2.3 million new cases diagnosed in 2020 [19]. The increase in incidence can be attributed to several factors, including improved screening practices, increased awareness, and lifestyle changes that influence risk factors.

Breast cancer can be categorized based on the age of onset, which has implications for the prognosis, treatment options, and biological behavior of the disease. Premenopausal breast cancer includes women diagnosed with breast cancer before the age of 50. Premenopausal breast cancer is often characterized by a more aggressive tumor biology, with a higher likelihood of being hormone receptor-negative and a greater incidence of triple-negative breast cancer (TNBC) [26]. Premenopausal breast cancer risk factors include genetic predispositions, particularly mutations in the *BRCA1* and *BRCA2* genes, along with lifestyle choices such as obesity, excessive alcohol consumption, and a lack of physical activity.

In contrast, postmenopausal breast cancer affects women aged 50 and older, who face different risk factors related to their health and medical history. Postmenopausal breast cancer is generally associated with a different set of risk factors, including hormonal changes related to menopause, age, and family history. The tumors in postmenopausal women are more likely to be hormone receptor-positive, which can influence treatment strategies, particularly the use of hormone therapy [27].

The age of onset significantly impacts the prognosis and treatment outcomes for breast cancer patients. Studies indicate that younger women tend to present with a more advanced disease at diagnosis, which can lead to poorer outcomes compared to older women [28]. Furthermore, the psychological and social implications of a breast cancer diagnosis can differ markedly between premenopausal and postmenopausal women, affecting their quality of life and treatment adherence.

Recent years have seen significant advancements in the prevention, diagnosis, and management of breast cancer, which have been pivotal in improving patient outcomes. These advancements encompass a range of areas, including screening techniques, treatment modalities, and an understanding of the disease’s biological mechanisms.

### Screening and Early Detection

Screening remains a cornerstone in the fight against breast cancer, with mammography being the most widely used method. However, despite the implementation of screening programs, the incidence of advanced-stage breast cancer has not decreased significantly over the past four decades. While screening has increased the detection of localized disease, it has not correspondingly reduced the incidence of de novo stage IV disease, indicating a complex interplay between screening practices and disease progression [29]. Similarly, Kirkpatrick et al. (2020) point out that socio-economic factors, including Medicaid expansion, have influenced the stage at which breast cancer is diagnosed, particularly among marginalized populations who are often present with a more advanced disease [30]. This underscores the necessity for effective screening and equitable access to healthcare services.

The treatment landscape for breast cancer has evolved significantly, particularly with the introduction of targeted therapies and immunotherapy. For instance, the advent of HER2-targeted therapies, such as trastuzumab, has markedly improved outcomes for patients with HER2-positive breast cancer since their introduction in the late 1990s [31]. Additionally, recent studies have explored the efficacy of metronomic chemotherapy regimens, such as those involving capecitabine, which have shown promise in treating metastatic triple-negative breast cancer [32]. These innovative treatment strategies are crucial, as they offer tailored approaches that can lead to better survival rates and quality of life for patients.

Research into the biological mechanisms of breast cancer has also advanced, providing insights that could lead to new therapeutic targets. For example, the roles of G-protein-coupled receptor 141 (GPR141) in breast cancer proliferation and metastasis have been elucidated, suggesting that targeting this receptor could offer new avenues for treatment [33]. Understanding the molecular pathways involved in breast cancer progression is essential for developing more effective therapies and improving patient outcomes. Regarding psychosocial factors and patient care, the psychological impact of a breast cancer diagnosis cannot be overlooked. Advanced stages of cancer often correlate with increased psychological stress, which can adversely affect treatment adherence and overall patient well-being [34]. Addressing these psychosocial factors is critical in the comprehensive management of breast cancer. Initiatives aimed at improving patient education and support systems can enhance early detection and treatment outcomes, as evidenced by studies indicating that an awareness of breast cancer symptoms significantly reduces delays in seeking care [35].

Breast cancer remains a complex disease with a significant global prevalence, affecting millions of women worldwide. The distinction between premenopausal and postmenopausal breast cancer is crucial for understanding the disease’s biology, risk factors, and treatment strategies. While advancements in research and clinical practice have improved outcomes for many patients, ongoing efforts are necessary to further enhance prevention, early detection, and treatment modalities. Continued research into the molecular underpinnings of breast cancer and the development of innovative therapies will be essential in reducing the burden of this disease.

## 3. Mechanisms of Resistance to Aromatase Inhibitors

Resistance to aromatase inhibitors in the treatment of breast cancer stems from a multifaceted interplay of intricate genetic variations, molecular pathways, and cellular interactions. These mechanisms collectively contribute to the tumor’s ability to evade the effects of therapy, highlighting the importance of further research to uncover these complexities and improve treatment outcomes for patients. Among the key factors are genetic and epigenetic alterations, such as mutations in the estrogen receptor gene (*ESR1*), which enable ligand-independent activation of the estrogen receptor. This allows tumors to continue growing despite aromatase inhibitor therapy. Additionally, modifications in critical signal transduction pathways, particularly the PI3K/AKT/mTOR and MAPK pathways, significantly enhance cell survival and proliferation [36].

**Figure 5:**
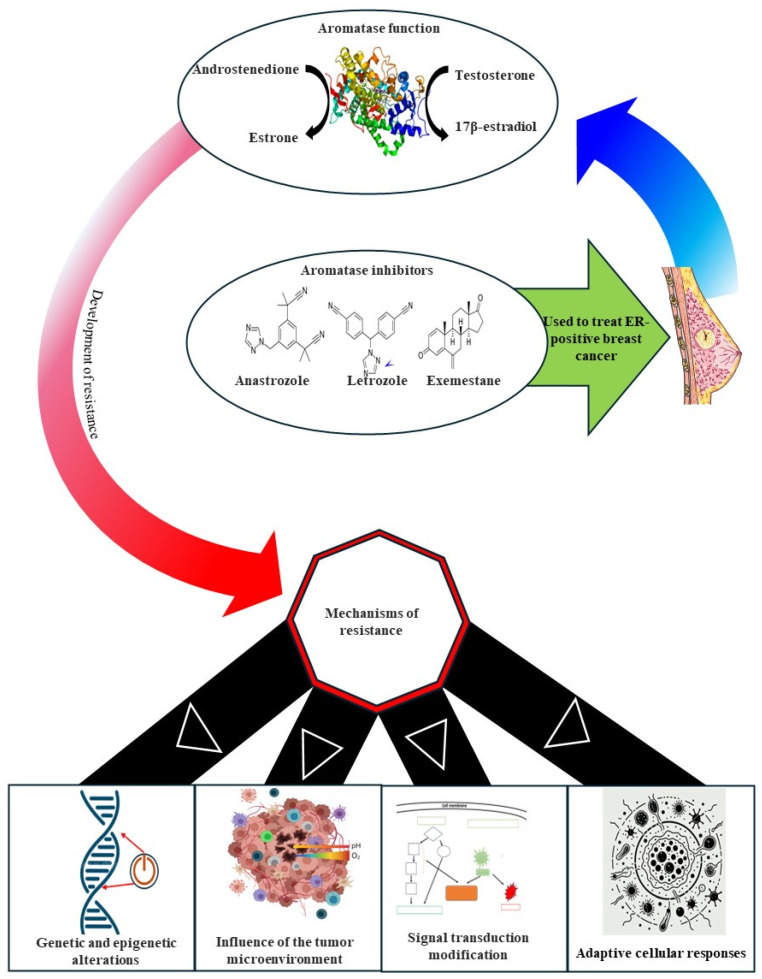
Mechanisms of resistance to aromatase inhibitors in ER-positive breast cancer. Mechanisms of resistance to aromatase inhibitors include genetic and epigenetic alterations, changes in the tumor microenvironment, alterations in specific signalling pathways and changes in cell behaviour or physiology that allow it to ensure their survival in response to treatment with aromatase inhibitors.

The tumor microenvironment, which consists of stromal cells, components of the extracellular matrix, hypoxia, and angiogenesis, all play a crucial role in resistance by promoting growth factor signaling and reducing drug efficacy. Furthermore, adaptive cellular responses, including autophagy and the epithelial–mesenchymal transition (EMT), enhance resistance by helping cancer cells endure therapeutic stress. A comprehensive understanding of these intricate mechanisms of resistance is vital for developing improved therapies to enhance patient outcomes, as illustrated in Figure 5.

### 3.1. Genetic and Epigenetic Alterations

A critical genetic alteration that drives resistance to aromatase inhibitors is the emergence of mutations in the *ESR1* gene. Notably, these mutations, especially those found in the ligand-binding domain (LBD), lead to ligand-independent activation of the estrogen receptor. This mechanism allows cancer cells to thrive and multiply, despite treatment with aromatase inhibitors [10,37,38]. Studies have shown that *ESR1* mutations are relatively rare in primary breast tumors but become prevalent in metastatic settings, especially after exposure to aromatase inhibitors [11,39]. For instance, a study indicated that the prevalence of *ESR1* mutations increased significantly in patients treated with aromatase inhibitors during the metastatic phase compared to those in the adjuvant phase (36% vs. 6%) [39]. Furthermore, these mutations are associated with a poor prognosis and reduced effectiveness of subsequent therapies, highlighting their role as a mechanism of acquired resistance [40,41].

In addition to direct mutations in *ESR1*, alterations in co-regulatory proteins that interact with the estrogen receptor can also contribute to resistance. These changes can affect the receptor’s transcriptional activity and its interactions with various signaling pathways [42]. For example, the dysregulation of coactivators and corepressors can lead to an altered response to hormonal therapies, thereby promoting resistance. The interplay between ESR1 mutations and co-regulatory proteins underscores the complexity of resistance mechanisms and suggests that targeting these interactions may provide therapeutic opportunities [43].

### 3.2. Signal Transduction Pathway Modifications—PI3K/AKT/mTOR Pathway

The phosphoinositide 3-kinase (PI3K)/AKT/mTOR signaling pathway is frequently implicated in the development of resistance to aromatase inhibitors. Activation of this pathway can promote cell survival and proliferation, counteracting the effects of endocrine therapies [44,45]. For instance, studies have demonstrated that inhibitors targeting components of this pathway can suppress aromatase activity in breast cancer cells, suggesting that aberrations in this signaling cascade may facilitate resistance [45]. Moreover, the presence of mutations in *PIK3CA*, a key component of the PI3K pathway, is associated with poor responses to aromatase inhibitors, indicating that this pathway’s activation is a critical factor in resistance [46].

### 3.3. MAPK Pathway

The mitogen-activated protein kinase (MAPK) pathway is another crucial signaling pathway that can be altered in breast cancer, contributing to resistance to aromatase inhibitors. Aberrant activation of this pathway can occur through various mechanisms, including mutations in upstream signaling components such as RAS and BRAF [47,48]. For example, the reactivation of the MAPK pathway has been shown to promote resistance to MEK inhibitors in colorectal cancer, suggesting that similar mechanisms may be at play in breast cancer [47]. In breast cancer, the interplay between the MAPK pathway and estrogen signaling can create a feedback loop that enhances tumor growth despite the presence of aromatase inhibitors [49].

### 3.4. Tumor Microenvironment Influences

#### 3.4.1. Roles of Stromal Cells and the Extracellular Matrix

The tumor microenvironment, including stromal cells and the extracellular matrix (ECM), plays a significant role in modulating the response to aromatase inhibitors. Interactions between cancer cells and the surrounding stroma can influence signaling pathways that promote resistance. For instance, stromal cells can secrete growth factors that enhance aromatase activity, thereby supporting tumor growth even in the presence of aromatase inhibitors. Additionally, the composition of the ECM can affect drug delivery and efficacy, further complicating treatment outcomes [46,50].

#### 3.4.2. Hypoxia and Angiogenesis

Hypoxia and the associated angiogenic responses within the tumor microenvironment can also contribute to resistance mechanisms. Hypoxic conditions can activate various signaling pathways that promote the survival and proliferation of cancer cells, including the upregulation of hypoxia-inducible factors (HIFs) [45]. These factors can enhance the expression of genes involved in angiogenesis, such as vascular endothelial growth factor (VEGF) and platelet-derived growth factor (PDGF), as well as metabolic adaptation-related genes, including metabolic enzymes like glucose transporter 1 (GLUT1). These mechanisms allow tumors to thrive despite therapeutic interventions [51]. The interplay between hypoxia, angiogenesis, and resistance to aromatase inhibitors highlights the importance of the tumor microenvironment in shaping treatment responses [50].

### 3.5. Adaptive Cellular Responses

#### 3.5.1. Autophagy

Autophagy, a cellular process that degrades and recycles cellular components, has been implicated in the development of resistance to aromatase inhibitors. In breast cancer, autophagy can provide a survival advantage under stress conditions, such as those induced by endocrine therapies. By facilitating the removal of damaged organelles and proteins, autophagy can help cancer cells adapt to the cytotoxic effects of aromatase inhibitors, thereby promoting resistance. Inhibiting autophagy has been proposed as a potential strategy to enhance the efficacy of aromatase inhibitors, suggesting that targeting this adaptive response may improve treatment outcomes [42,52].

#### 3.5.2. Epithelial–Mesenchymal Transition (EMT)

The epithelial–mesenchymal transition (EMT) is another adaptive response that can contribute to resistance to aromatase inhibitors. The EMT is characterized by the loss of epithelial characteristics and the acquisition of mesenchymal traits, which can enhance cell motility and invasiveness [45]. This transition is often driven by various signaling pathways, including those activated by growth factors and cytokines present in the tumor microenvironment [50]. The induction of the EMT can lead to a more aggressive tumor phenotype that is less responsive to endocrine therapies such as tamoxifen, fulvestrant, or exemestane, underscoring the need for strategies that target this process to overcome resistance [53].

## 4. Radiogenomics: An Integrative Approach

Radiogenomics is an emerging interdisciplinary field that integrates imaging and genomic data to enhance our understanding of cancer biology and improve clinical outcomes. This synthesis will explore the definition and scope of radiogenomics, the imaging techniques employed, and the genomic profiling methods utilized, supported by the relevant literature.

### 4.1. Combining Imaging and Genomic Data

Radiogenomics refers to the integration of radiological imaging features with genomic data from tumors, creating a comprehensive approach to understanding cancer. This integration allows for the identification of correlations between imaging phenotypes and genetic alterations, which can provide insights into tumor behavior and treatment responses [54,55]. The field has evolved significantly, moving beyond traditional imaging to include advanced techniques such as functional imaging and radiomics, which extract quantitative data from images. This evolution is crucial for developing predictive models that can guide personalized treatment strategies in oncology [56,57,58].

**Figure 6:**
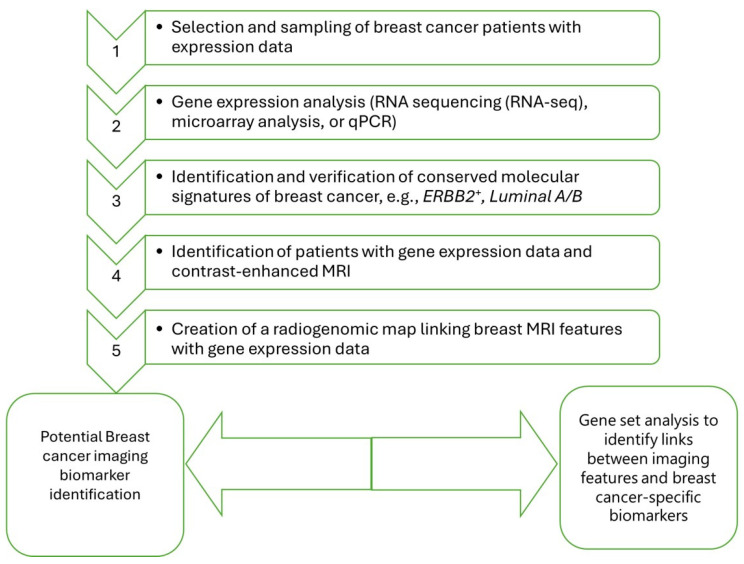
Summary of radiogenomic steps used to identify potential breast cancer imaging biomarkers. This process aims to identify potential imaging biomarkers for breast cancer by integrating gene expression data with MRI findings. It starts with the selection of breast cancer patients who have corresponding gene expression data. A gene expression analysis is then performed to identify and validate molecular signatures such as *ERBB2+* or *Luminal A/B*. To further refine this approach, Figure 6 illustrates the radiogenomic workflow. The initial step involves identifying patients with both gene expression data and MRI scans. Once these patients are selected, a radiogenomic map is generated to establish associations between MRI features and gene expression profiles.

The primary objectives of radiogenomics include improving the diagnostic accuracy, predicting treatment responses, and understanding tumor heterogeneity [57,58]. By combining imaging and genomic data, clinicians can better stratify patients based on their individual tumor characteristics, leading to more tailored therapeutic approaches [54,55]. The potential benefits of this integrative approach include enhanced prognostic capabilities, improved patient outcomes, and the ability to monitor treatment efficacy in real-time. Furthermore, radiogenomics may facilitate non-invasive tumor genotyping, reducing the need for invasive biopsies [59]. As illustrated in Figure 6, breast cancer patient are selected and sampled. Various techniques, such as RNA sequencing, are employed so as to assist in the identification of conserved molecular signatures, e.g., *Luminal A/B*. Imaging techniques such as MRI are concurrently applied to similar samples. The generated result will be used to create a linkage map using the MRI and gene expression data. This two-pronged approach ultimately result in the generation of reliable data in this specific circumstance for breast cancer-specific biomarkers.

By integrating imaging and molecular data, this methodology holds significant promise for uncovering novel imaging biomarkers. These biomarkers could enhance the accuracy of breast cancer diagnosis, improve risk stratification, and inform personalized treatment planning, ultimately contributing to better patient outcomes.

### 4.2. Imaging Techniques in Radiogenomics—MRI, CT, PET, and Mammography

**Figure 7:**
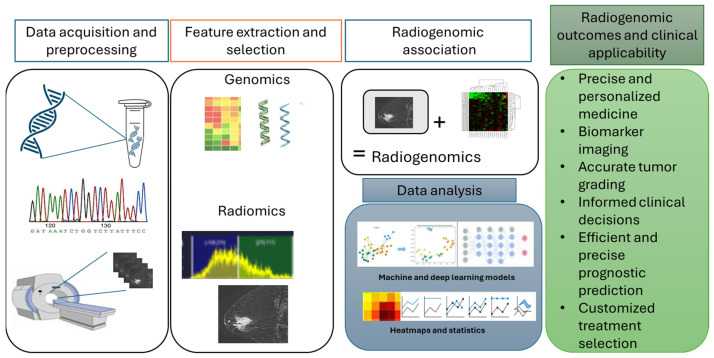
Workflow for a radiogenomic analysis in breast cancer imaging biomarker discovery. This diagram illustrates a radiogenomic workflow for discovering imaging biomarkers in breast cancer, beginning with the acquisition and preprocessing of genomic and imaging data, followed by the extraction of relevant gene expression and imaging features. Genomic and radiomic data are then integrated to establish radiogenomic associations, linking imaging characteristics with molecular profiles. Machine learning models analyze these associations, generating heatmaps and statistical insights. The workflow’s outcomes enable personalized medicine applications, including biomarker-based imaging, accurate tumor grading, and customized treatment planning, supporting precision in breast cancer diagnosis and therapy.

Radiogenomics utilizes various imaging modalities to provide insights into tumor characteristics. Magnetic resonance imaging (MRI) is notable for its exceptional soft tissue contrast, allowing for detailed assessments of the tumor morphology and heterogeneity. Other techniques, such as computed tomography (CT), positron emission tomography (PET), and mammography, each offer unique benefits. CT scans deliver rapid cross-sectional images, PET scans reveal metabolic activity within tumors, and mammography is essential for early breast cancer detection. Together, these imaging methods enhance our understanding of cancer biology and support personalized treatment strategies [60,61]. Building on these insights, Figure 7 presents a radiogenomic workflow aimed at identifying image biomarkers in breast cancer by integrating genomic and imaging data to enhance personalized medicine. The process begins with data acquisition and preprocessing, followed by the extraction of gene expression and radiomic features. By establishing connections between these features, the workflow utilizes machine learning models to derive insights through heatmaps and statistical analysis. This methodology enhances the precision of the breast cancer diagnosis and treatment, with applications in biomarker-based imaging, tumor grading, and tailored treatment planning.

Table 1 summarizes key biomarkers relevant to aging and tumor grading, along with their applications in imaging techniques such as MRI, CT, and PET. These biomarkers serve as critical indicators in facilitating personalized approaches to cancer diagnosis and treatment. MRI excels in capturing informative data concerning soft tissue, providing detailed assessments of the soft tissue morphology and functional dynamics of biomarkers such as *Ki-67* and VEGF. CT delivers a rapid visualization of anatomical changes, aiding in the structural assessment of markers like p53 and MMPs. PET stands out for its ability to quantify metabolic activity associated with markers like GLUT1 and VEGF, enhancing the precision of tumor characterization. By integrating these insights, imaging techniques contribute significantly to biomarker-based imaging, tumor grading, and tailored treatment planning, enabling better diagnostic and therapeutic strategies and ultimately advancing the field of personalized medicine. CT is widely used for its ability to provide detailed anatomical information, while PET is valuable for evaluating metabolic activity within tumors [62,63]. The integration of these imaging modalities enhances the overall understanding of tumor biology and supports the development of radiogenomic models [60,62].

### 4.3. Functional Imaging

Functional imaging techniques, such as dynamic contrast-enhanced MRI (DCE-MRI) and PET/CT with specific tracers, play a pivotal role in radiogenomics. DCE-MRI allows for the assessment of tumor perfusion and vascular permeability, providing insights into the tumor microenvironment and response to therapy [65]. Similarly, PET/CT can be utilized with various tracers to evaluate metabolic processes and receptor expression, further elucidating the relationship between imaging features and genomic data. These advanced imaging techniques are essential for capturing the dynamic nature of tumors and their interactions with treatment.

### 4.4. Genomic Profiling Methods

#### 4.4.1. Next-Generation Sequencing (NGS)

Next-generation sequencing (NGS) has revolutionized genomic profiling by enabling a comprehensive analysis of tumor genomes at an unprecedented scale. NGS allows for the identification of mutations, copy number variations, and other genomic alterations that may influence tumor behavior and treatment responses. The integration of NGS data with imaging features can enhance the predictive power of radiogenomic models, allowing for more accurate assessments of tumor characteristics and treatment outcomes.

#### 4.4.2. Gene Expression Profiling

Gene expression profiling is another critical component of radiogenomics, providing insights into the functional status of tumors. By analyzing the expression levels of specific genes, researchers can identify molecular signatures associated with tumor aggressiveness, treatment resistance, and patient prognosis. This information can be correlated with imaging features to develop robust predictive models that guide clinical decision-making [66,67].

#### 4.4.3. Liquid Biopsy and Circulating Tumor DNA (ctDNA)

Liquid biopsy techniques, particularly the analysis of circulating tumor DNA (ctDNA), offer a non-invasive method for genomic profiling. ctDNA can provide real-time insights into tumor dynamics, including the detection of mutations and alterations associated with treatment resistance [59]. The integration of a ctDNA analysis with imaging data enhances the understanding of tumor heterogeneity and allows the monitoring of treatment responses over time. This approach is particularly valuable in the context of personalized medicine, where timely adjustments to treatment strategies can significantly impact patient outcomes [68].

## 5. Radiogenomic Landscape in Metastatic Endocrine-Positive Breast Cancer

### 5.1. Correlation Between Imaging Features and Genomic Alterations

The integration of radiogenomics in metastatic endocrine-positive breast cancer represents a transformative approach that links imaging phenotypes with genomic alterations, providing a deeper understanding of tumor behavior and treatment resistance. This emerging field aims to identify non-invasive radiographic biomarkers that correlate with specific genomic changes, thereby enhancing the precision of treatment strategies.

#### 5.1.1. Identifying Radiographic Biomarkers of Resistance

Radiogenomics has the potential to uncover critical insights into the mechanisms of resistance to endocrine therapies in metastatic breast cancer. Imaging modalities such as multi-parametric MRI and PET/CT, alongside advanced techniques like radiomics, have been pivotal in identifying radiographic features that predict treatment resistance. For instance, specific patterns observed in radiomic texture analysis of bone metastases have been correlated with *ESR1* mutations, which are known to confer resistance to aromatase inhibitors [69]. These mutations in *ESR1* are particularly significant, as they can alter the receptor’s conformation, leading to a diminished response to endocrine therapies [69]. Moreover, alterations in the PI3K/AKT/mTOR signaling pathway, which are frequently observed in endocrine-resistant tumors, have been linked to specific imaging characteristics. Studies have shown that tumors harboring *PIK3CA* mutations often present with distinct radiographic features that can be detected through advanced imaging techniques [70]. For example, the presence of *PIK3CA* mutations is associated with a more aggressive tumor phenotype, which can be visualized as increased metabolic activity on PET scans [71]. Recent research has also highlighted the importance of a circulating DNA (ctDNA) tumor analysis in conjunction with imaging. ctDNA can provide insights into the genomic landscape of tumors, allowing the identification of mutations that may not be detectable through traditional biopsy methods. This combination of imaging and genomic analysis can enhance the understanding of tumor heterogeneity and resistance mechanisms, ultimately guiding more personalized treatment approaches [72,73].

#### 5.1.2. Advancements in Imaging Techniques

The emergence of advanced imaging techniques has greatly enhanced the radiogenomic field. Radiomics, which extracts numerous quantitative features from medical images, has demonstrated potential in identifying imaging biomarkers linked to specific genomic changes. For instance, studies have demonstrated that radiomic features can predict the presence of *PIK3CA* mutations and other genomic alterations in breast cancer patients [74,75]. These include alterations such as in *ERBB2* (HER2), which is frequently amplified in aggressive breast cancer subtypes, or PTEN (phosphatase and tensin homolog), a tumor suppressor gene that, when deleted or mutated, can lead to the activation of the PI3K/AKT signaling pathway. Additionally, *TP53* mutations, which are common in various cancers and often correlate with a more aggressive disease, can be predicted through radiomic features as well. These genomic alterations are important in regulating cell cycle progression, apoptosis, and cellular stress responses, and their presence can significantly impact tumor behavior [75]. Radiomic features may also correlate with the expression of proteins like pAkt (phosphorylated Akt) and pERK (phosphorylated extracellular signal-regulated kinase), which are key players in cell survival, proliferation, and invasion. For example, higher levels of *pAkt* or *pERK* expression, which are often associated with resistance to therapies, may be visualized through texture and intensity patterns in imaging data. These correlations further strengthen the ability of radiomics to serve as a non-invasive tool for identifying specific molecular and genomic alterations in breast cancer [74,75]. These features can include texture, shape, and intensity variations in imaging data, which correlate with underlying biological processes. The integration of artificial intelligence (AI) and machine learning algorithms in radiomics is leading to a more accurate and efficient analysis of imaging data. AI-driven models can examine complex imaging datasets to identify patterns that may indicate specific genomic alterations. This advancement facilitates the early detection of resistance to endocrine therapies [74,75,76,77].

#### 5.1.3. Clinical Implications and Future Directions

The clinical implications of radiogenomics in metastatic endocrine-positive breast cancer are profound. By identifying radiographic biomarkers of resistance, clinicians can tailor treatment strategies based on the individual genomic profile of the tumor. For instance, patients with *PIK3CA* mutations may benefit from the addition of targeted therapies, such as alpelisib, to their endocrine treatment regimen, which has been shown to improve progression-free survival [78,79]. Moreover, the ongoing research into the correlations between imaging features and genomic alterations emphasizes the need for routine genomic testing in clinical practice. As the understanding of the genomic landscape of breast cancer evolves, integrating genomic data with imaging findings will be crucial for optimizing treatment strategies and improving patient outcomes [80,81]. In conclusion, the radiogenomic landscape of metastatic endocrine-positive breast cancer is an exciting and rapidly evolving field that holds the potential to revolutionize the management of this disease. By bridging the gap between imaging and genomics, clinicians can gain valuable insights into tumor behavior and resistance mechanisms, ultimately leading to more personalized and effective treatment approaches.

#### 5.1.4. Implications for Clinical Practice

These clinical examples highlight the transformative potential of radiogenomics in the management of metastatic endocrine-positive breast cancer. By correlating imaging features with genomic alterations, clinicians can gain valuable insights into tumor behavior and resistance mechanisms. This integration allows the identification of patients who may benefit from alternative therapies, thereby improving treatment outcomes and minimizing unnecessary exposure to ineffective treatments. Moreover, the advancements in imaging technologies, such as multi-parametric MRI and PET/CT, combined with radiomic analysis, enhance the ability to detect subtle changes in tumor characteristics that may indicate resistance to therapy [82]. As personalized medicine continues to evolve, incorporating genomics and imaging features into a unified treatment strategy could provide clinicians with a more comprehensive understanding of tumor dynamics, leading to better-targeted therapies. For instance, combining genomic data from NGS (next-generation sequencing) with functional imaging techniques like dynamic contrast-enhanced MRI could allow clinicians to detect early signs of tumor progression or treatment failure before clinical symptoms appear, improving timely interventions [83]. As the field of radiogenomics continues to evolve, more robust predictive models are expected to emerge, further refining the approach to personalized medicine in oncology.

The integration of radiogenomics into clinical practice represents a significant advancement in the management of metastatic endocrine-positive breast cancer. The case studies discussed illustrate how correlating imaging features with genomic alterations can guide treatment decisions and improve patient outcomes. With ongoing research into how radiogenomics can predict resistance mechanisms and optimize therapeutic regimens, there is great potential to revolutionize the treatment landscape for breast cancer patients. For example, the development of radiogenomic models that integrate genomic data, imaging features, and patient clinical profiles could help predict the likelihood of resistance to multiple therapy classes, enabling proactive and more individualized treatment regimens [84]. As research in this field progresses, the potential for radiogenomics to revolutionize cancer care becomes increasingly evident.

### 5.2. Patterns of Metastasis and Genomic Profiles

#### 5.2.1. Common Metastatic Sites: Bone, Liver, Lung, and Brain

In metastatic endocrine-positive breast cancer, the patterns of metastasis reveal a distinct hierarchy of common sites, with the bone being the most prevalent, followed by the liver, lung, and brain. Each of these sites exhibits unique genomic signatures that influence the behavior and progression of metastatic disease.

#### 5.2.2. Bone Metastases

Bone metastases are the most frequent site of spread of endocrine-positive breast cancer and are often associated with specific genomic alterations. Notably, *ESR1* mutations have been linked to bone metastases. Research indicates that *ESR1* mutations can enhance cell adhesion properties, promoting the establishment of metastases in the bone microenvironment [85]. A study by Bartels reported that approximately 12% of bone metastases from breast cancer exhibited *ESR1* mutations, with this frequency rising to 14% in estrogen receptor α-positive cases [86]. The presence of these mutations is significant, as they are associated with resistance to endocrine therapies, complicating treatment strategies.

#### 5.2.3. Liver Metastases

Liver metastases in patients with breast cancer frequently show alterations in the PI3K/AKT/mTOR signaling pathway, with *PIK3CA* mutations being particularly prominent. Research indicates that these mutations are notably prevalent in the liver metastases associated with the luminal subtype of breast cancer. This enrichment of *PIK3CA* mutations suggests a potential underlying mechanism that may lead to resistance to conventional endocrine therapies, complicating treatment outcomes for these patients [87]. Additionally, a study found that *ESR1* variants were more common in liver metastasis samples compared to primary breast samples, with a mutation rate of 27% observed in liver metastases [38]. This suggests that liver metastases may harbor distinct genomic profiles that contribute to their aggressive nature and therapeutic resistance.

#### 5.2.4. Lung Metastases

The lung is another common site for the metastatic spread of breast cancer. While the specific genomic alterations associated with lung metastases are less well-defined compared to bone and liver metastases, the presence of *PIK3CA* mutations has been noted. The mutational landscape in lung metastases often reflects the overall genomic profile of the primary tumor, with variations depending on the intrinsic subtype of breast cancer [64]. The complexity of lung metastases is compounded by the potential for additional mutations that may arise during the metastatic process, further complicating treatment approaches.

#### 5.2.5. Brain Metastases

Brain metastases in breast cancer patients are particularly concerning and are often associated with *HER2* amplification or *BRCA1/2* mutations. The presence of these mutations can significantly influence the treatment landscape, as HER2-positive tumors may respond to targeted therapies such as trastuzumab, while *BRCA* mutations may indicate susceptibility to PARP inhibitors [88]. The genomic profile of brain metastases tends to differ from that of other metastatic sites, highlighting the need for tailored therapeutic strategies. For instance, a study by Aftimos noted that mutations in *TP53*, *PIK3CA*, and *ESR1* were prevalent in both primary tumors and matched brain metastases, emphasizing the importance of understanding the genomic landscape in managing these patients [38,87].

### 5.3. Imaging Modalities and Their Roles

Imaging modalities such as bone scintigraphy, liver MRI, and brain MRI play a crucial role in characterizing these metastatic sites. These imaging techniques provide critical insights into the biological aggressiveness of metastatic tumors and can help identify specific patterns of spread that correlate with the underlying genomic alterations. For example, advanced imaging techniques can detect changes in metabolic activity and tumor morphology, which may indicate the presence of specific mutations or alterations in signaling pathways [89].

The patterns of metastasis in endocrine-positive breast cancer are intricately linked to specific genomic profiles that influence treatment resistance and disease progression. Understanding the unique characteristics of common metastatic sites, such as the bone, liver, lung, and brain, along with their associated genomic alterations, is essential for developing personalized therapeutic strategies. As research in the field of metastatic endocrine-positive breast cancer continues to advance, the fusion of genomic data with cutting-edge imaging techniques holds great promise. This integration may significantly enhance our capability to predict how individual patients will respond to various treatments, ultimately leading to improved outcomes and a better quality of life for those affected by this complex disease. By leveraging both genetic insights and detailed imaging, we can develop more personalized treatment plans tailored to the unique characteristics of each patient’s cancer [90].

## 6. Genomic Signatures Associated with Metastatic Patterns in Endocrine-Positive Breast Cancer

Recent research has highlighted several key genomic alterations associated with metastatic patterns in endocrine-positive breast cancer, which are crucial for optimizing treatment strategies. ESR1 mutations, which are common in patients who develop bone metastasis, enhance the adhesive properties of cancer cells, facilitating their colonization in the bone microenvironment, and are observed in approximately 30–40% of patients with metastatic endocrine-positive breast cancer, particularly those with hormone receptor-positive breast cancer [91]. TP53 mutations, which are linked to more aggressive tumor behavior, are associated with a higher likelihood of visceral metastases, particularly in the liver and brain, due to the loss of tumor suppressor function and increased genomic instability, with a prevalence of approximately 20–30% in patients with metastatic breast cancer [92]. *FGFR1* amplification, a poor prognostic marker, is implicated in lung metastasis, promoting the epithelial–mesenchymal transition (EMT), is found in 5–10% of patients with endocrine-positive breast cancer, and is often associated with a poor prognosis [93]. CDK4/6 pathway alterations, particularly amplifications of CDK4/6, are linked to resistance to CDK4/6 inhibitors, which are commonly used to treat ER-positive breast cancer, reducing the efficacy of these inhibitors, with a prevalence of 10–15% in patients, especially those with lung metastases or resistance to standard therapies [94,95]. The integration of radiogenomics allows clinicians to correlate imaging features with specific genomic alterations, aiding in patient stratification and identifying those who may benefit from personalized therapies based on their unique genomic profiles to ultimately improve treatment outcomes, with radiomic patterns associated with *ESR1*, *TP53*, or *CDK4*/*6* alterations offering insights into the treatment response.

### 6.1. ESR1 Mutations and Bone Metastasis

ESR1 mutations are frequently observed in patients with endocrine-positive breast cancer and are particularly associated with a propensity for bone metastasis. These mutations often lead to a more indolent disease course, allowing tumors to establish in the bone microenvironment [91]. Research indicates that ESR1 mutations can enhance the adhesive properties of cancer cells, facilitating their colonization in bone tissue [96]. A study demonstrated that patients with ESR1 mutations exhibited a higher incidence of bone metastases, underscoring the need for vigilant monitoring and tailored treatment approaches for this subgroup [91].

### 6.2. TP53 Mutations and Aggressive Metastatic Behavior

In contrast, TP53 mutations are linked to more aggressive forms of breast cancer and correlate with a higher likelihood of liver and brain metastases. These mutations are associated with a loss of tumor suppressor function, leading to increased genomic instability and a more aggressive tumor phenotype [92,97]. Studies have shown that patients with *TP53* mutations often present with an advanced disease at diagnosis, which is reflected in the propensity for visceral metastases, particularly in the liver and brain [97]. The presence of *TP53* mutations necessitates a more aggressive treatment approach, as these tumors are less responsive to standard endocrine therapies [97,98].

### 6.3. FGFR1 and CDK4/6 Pathway Alterations in Lung Metastasis

Alterations in the fibroblast growth factor receptor 1 (FGFR1) and cyclin-dependent kinase 4/6 (CDK4/6) pathways have been implicated in lung metastasis. FGFR1 amplification is a known poor prognostic marker of breast cancer and is associated with enhanced metastatic potential [93]. FGFR1 signaling promotes the epithelial–mesenchymal transition (EMT), a critical process that facilitates metastasis to the lungs [99]. Additionally, CDK4/6 inhibitors, such as palbociclib, are commonly used to treat ER-positive breast cancer; however, resistance can develop due to alterations in the CDK4/6 pathway [95]. The presence of CDK4/6 amplifications has been shown to reduce sensitivity to these inhibitors, complicating treatment regimens for patients with lung metastases [94].

### 6.4. Radiogenomics: A Tool for Risk Stratification and Personalized Therapy

The integration of radiogenomics offers a promising avenue for stratifying patients based on their metastatic patterns and associated genomic profiles. By correlating imaging features with genomic alterations, clinicians can identify patients at higher risk for aggressive disease and tailor therapeutic interventions accordingly. For instance, radiomic analysis of imaging data can reveal patterns indicative of specific mutations, such as those in *ESR1* or *TP53*, allowing for more personalized treatment strategies [100]. This approach not only enhances the understanding of tumor behavior but also facilitates the timely adjustment of treatment plans based on the evolving genomic landscape of metastatic disease.

Specific genomic alterations play a critical role in determining the metastatic patterns observed in endocrine-positive breast cancer. *ESR1* mutations are associated with bone metastasis, while TP53 mutations correlate with aggressive liver and brain metastases. Additionally, alterations in the FGFR1 and CDK4/6 pathways are implicated in lung metastasis. The emerging field of radiogenomics holds significant promise for improving patient stratification and personalizing therapeutic interventions based on the unique genomic profiles associated with metastatic patterns. As research continues to advance, the integration of genomic data with clinical practice will be essential for optimizing the treatment outcomes of patients with metastatic endocrine-positive breast cancer.

## 7. Radiogenomic Signatures of Resistance

Radiogenomics, which encompasses the integration of imaging phenotypes with genomic data, has established itself as a vital field in advancing our understanding of the complexities surrounding breast cancer, particularly in relation to resistance to endocrine therapies. This synthesis aims to explore two fundamental aspects of radiogenomics: the imaging biomarkers associated with *ESR1* mutations and the radiogenomic patterns linked to alterations in the PI3K/AKT/mTOR pathway.

### 7.1. Imaging Biomarkers Linked to ESR1 Mutations

ESR1 mutations are recognized as significant contributors to resistance to endocrine therapies, especially aromatase inhibitors, in estrogen receptor-positive breast cancer. Recent studies have identified imaging biomarkers that correlate with these mutations, enhancing the potential for non-invasive diagnostics. For instance, bone metastases in patients with *ESR1* mutations often exhibit sclerotic features on computed tomography (CT) scans, while positron emission tomography (PET) scans reveal specific metabolic patterns indicative of aggressive disease [67]. Furthermore, magnetic resonance imaging (MRI) has shown increased heterogeneity in the imaging characteristics of both primary tumors and metastases in patients who have undergone multiple lines of therapy, suggesting that these mutations may lead to more complex tumor biology [101,102]. The ability to detect ESR1 mutations through imaging could facilitate earlier interventions with alternative therapies, such as selective estrogen receptor degraders (SERDs), which have shown promise in overcoming resistance mechanisms [103]. The integration of radiogenomic approaches allows for a more nuanced understanding of tumor biology, where imaging features can reflect underlying genetic alterations, thus aiding in personalized treatment strategies [100]. For example, studies have demonstrated that specific MRI characteristics can predict the presence of *ESR1* mutations, thereby guiding therapeutic decisions [104].

### 7.2. Case Studies and Clinical Examples

The integration of radiogenomics into the management of metastatic endocrine-positive breast cancer has opened new avenues for personalized treatment strategies. This section discusses notable clinical examples that illustrate the utility of radiogenomic data in predicting treatment responses and guiding therapeutic decisions.

#### 7.2.1. Clinical Example 1: Monitoring the Treatment Response with PET/CT Imaging

In a notable case, a patient with metastatic endocrine-positive breast cancer underwent PET/CT imaging to monitor the treatment response. Genomic sequencing revealed an ESR1 mutation, which is known to correlate with resistance to first-line endocrine therapy. This mutation alters the estrogen receptor’s function, leading to reduced sensitivity to aromatase inhibitors, a common treatment for hormone receptor-positive breast cancer [105]. A radiomic analysis of the liver metastasis demonstrated increased heterogeneity in imaging features, indicative of treatment-resistant disease. Specifically, the texture analysis revealed distinct patterns that correlated with the presence of the ESR1 mutation, suggesting that the tumor’s biological behavior was more complex than initially anticipated [75]. Furthermore, the mutation was associated with a decreased response to selective estrogen receptor degraders (SERDs), highlighting the need for alternative hormonal therapies or combination treatments. For example, the use of the SERD drug, fulvestrant, in combination with CDK4/6 inhibitors like palbociclib has shown promise in overcoming resistance caused by ESR1 mutations, offering an effective alternative approach. This case underscores the potential of integrating radiogenomic data to identify patients who may not respond to standard therapies, thereby guiding clinicians to consider alternative treatment options earlier in the disease course [106].

#### 7.2.2. Clinical Example 2: Targeted Therapy for PI3K Pathway Alterations

Another clinical study involved a patient with identified alterations in the PI3K pathway. Upon a genomic analysis, mutations in the *PIK3CA* gene were detected, which are often associated with endocrine resistance in breast cancer [57]. The patient was subsequently treated with a targeted therapy that inhibits the PI3K pathway, leading to a significant reduction in metabolic activity, as observed on FDG-PET scans. This reduction in metabolic activity is a direct indicator of treatment efficacy, demonstrating how imaging can track the real-time response to targeted therapies. The ability to visualize changes in tumor metabolism through imaging not only provides immediate feedback on treatment effectiveness but also allows for timely adjustments in therapeutic strategies. Moreover, the combination of PI3K pathway inhibitors with immune checkpoint inhibitors has shown promising results in overcoming resistance and improving patient outcomes. For instance, studies have shown that combining the PI3K inhibitor alpelisib with the immune checkpoint inhibitor pembrolizumab can improve therapeutic responses in patients with *PIK3CA* mutations, demonstrating the power of combining targeted therapy with immunotherapy to combat resistance [107]. In this case, the integration of radiogenomic data facilitated a more personalized approach, optimizing the patient’s treatment plan based on the specific genomic alterations present in their tumor [108,109].

### 7.3. Radiogenomic Patterns of PI3K/AKT/mTOR Pathway Alterations

Alterations in the PI3K/AKT/mTOR signaling pathway are prevalent in estrogen receptor-positive breast cancers, particularly in advanced or metastatic cases. Radiogenomic studies have identified distinct imaging features associated with these alterations. For instance, increased fluorodeoxyglucose (FDG) uptake on PET scans is often observed in tumors with PI3K pathway mutations, indicating the heightened metabolic activity typical of aggressive cancers [110]. Additionally, liver metastases harboring these mutations frequently exhibit high contrast enhancement on dynamic contrast-enhanced MRI (DCE-MRI) attributed to increased vascularity [67,101]. Targeting these genomic alterations with specific agents, such as alpelisib, a PI3K inhibitor, has demonstrated efficacy in clinical settings, underscoring the importance of imaging in monitoring therapeutic responses [111]. The ability to correlate imaging findings with genomic data not only enhances our understanding of tumor behavior but also aids in predicting treatment outcomes. For example, studies have shown that radiogenomic signatures can reveal intratumor heterogeneity associated with biological functions, which in turn correlates with patient survival [112,113]. This relationship emphasizes the potential of radiogenomics to inform treatment strategies and improve patient outcomes through tailored therapies based on individual tumor characteristics.

## 8. Clinical Implications and Applications

The integration of radiogenomic data into clinical practice profoundly influences the management of breast cancer by enabling personalized treatment, predicting outcomes, and monitoring disease progression. By connecting advanced imaging techniques with molecular biology, radiogenomics offers valuable insights into tumor behavior and enhances the precision of therapeutic interventions. This evolving approach facilitates real-time decision-making and extends its impact to broader oncology practices, marking a new era in precision medicine. This section will analyze the clinical implications of the findings, focusing on personalized treatment strategies that cater to individual patient profiles. Additionally, it will discuss methods for monitoring disease progression and assessing treatment responses to enhance patient outcomes and optimize therapeutic interventions.

### 8.1. Personalized Treatment Strategies

The advent of radiogenomics allows clinicians to tailor therapies based on the unique genetic and imaging profiles of individual tumors. For instance, the identification of specific biomarkers associated with tamoxifen resistance, such as SOX2 and AGR2, can guide the selection of alternative therapies for estrogen receptor-positive (ER+) breast cancer patients who exhibit resistance to standard treatments [114]. Additionally, the use of steroid receptor coactivator (SRC) inhibitors in combination with tamoxifen has shown potential in overcoming resistance mechanisms, highlighting the importance of integrating genomic insights into treatment planning [115].

Combination therapies that target multiple pathways are becoming increasingly relevant in managing resistance. For example, the combination of histone deacetylase (HDAC) inhibitors with tamoxifen has been explored as a strategy to enhance the efficacy of endocrine therapy in hormone-resistant breast cancer [116]. Furthermore, the use of novel agents such as palbociclib, a cyclin-dependent kinase 4/6 inhibitor, has demonstrated efficacy when combined with aromatase inhibitors in ER-positive breast cancer, emphasizing the role of targeted therapies in personalized treatment regimens [117].

The field of radiogenomics is also advancing precision radiotherapy by enabling the integration of genomic and imaging data to optimize radiation treatment plans. For instance, radiogenomic approaches are employed to identify biomarkers predictive of radiation sensitivity and resistance, facilitating the development of personalized radiotherapy protocols. Clinical studies have demonstrated the potential of these approaches in enhancing tumor control while minimizing radiation-induced toxicity to healthy tissues [118]. Recent advancements include the application of artificial intelligence in radiogenomics to predict patient-specific responses to radiotherapy. For example, deep learning models trained on imaging and genomic datasets have been used to forecast lung cancer and glioblastoma radiotherapy outcomes, further refining personalized treatment strategies [55].

### 8.2. Predictive and Prognostic Value

Radiogenomic signatures can serve as powerful predictive tools for the treatment response. For instance, studies have shown that specific imaging features correlate with the presence of genomic alterations that predict resistance to therapies like tamoxifen [119]. By employing advanced imaging techniques, clinicians can identify patients who are less likely to benefit from standard treatments, thereby facilitating the timely initiation of alternative therapeutic strategies.

The prognostic value of radiogenomic profiles is becoming increasingly recognized. For example, high levels of Stathmin 1 (*STMN1*) or oncoprotein 18 are associated with a poor prognosis and chemotherapy resistance in various cancers, including breast cancer, indicating that specific biomarkers can inform clinicians about the likely course of the disease and guide treatment decisions [120]. Furthermore, the identification of unique radiogenomic patterns may help stratify patients into different risk categories, allowing for more tailored follow-up and management strategies.

In oncology, radiogenomic profiles are increasingly being used in clinical settings to predict tumor behavior and tailor treatment strategies. For example, radiogenomic data have been employed to characterize glioblastomas, revealing imaging–genomic associations that provide insights into tumor aggressiveness and therapeutic vulnerabilities. Such applications underscore the expanding role of radiogenomics in improving precision oncology across diverse cancer types [57]. More recently, researchers have leveraged radiogenomic signatures to predict immunotherapy responses in non-small cell lung cancer (NSCLC), identifying specific imaging biomarkers associated with PD-L1 expression. These advancements are enabling clinicians to stratify patients more effectively and optimize therapeutic decisions [51].

### 8.3. Monitoring Disease Progression and the Treatment Response

Sequential imaging combined with a genomic analysis is crucial for monitoring disease progression and the treatment response. By regularly assessing imaging biomarkers alongside genomic data, clinicians can gain insights into how tumors evolve and adapt to therapies [119,121]. This approach not only aids in evaluating the effectiveness of ongoing treatments but also helps in identifying early signs of resistance, allowing for timely modifications to the treatment plan.

Detecting resistance and disease progression early is vital for improving patient outcomes. Radiogenomic approaches can facilitate the identification of changes in tumor characteristics that signal emerging resistance, such as alterations in metabolic activity observed through PET imaging [121]. By integrating these findings with genomic data, clinicians can develop strategies to preemptively address resistance, potentially improving the efficacy of treatment regimens and enhancing overall survival rates.

Advances in radiogenomic technologies have also enabled the development of dynamic monitoring tools that combine longitudinal imaging and genomic data. These tools are being used in clinical trials to assess the real-time efficacy of novel therapies and adapt treatment strategies accordingly. Such integrative approaches are particularly valuable in addressing tumor heterogeneity and ensuring that therapeutic interventions remain effective throughout treatment [118]. Recent studies have demonstrated the utility of radiogenomic biomarkers in monitoring the response to CAR-T cell therapies in hematologic malignancies, providing a non-invasive method to evaluate treatment efficacy and detect relapses [122].

The integration of radiogenomic data into clinical practice offers a transformative approach to breast cancer management. By personalizing treatment strategies, leveraging predictive and prognostic insights, and enhancing monitoring capabilities, clinicians can significantly improve patient outcomes and navigate the complexities of treatment resistance in breast cancer.

## 9. Future Directions and Research

The rapid evolution of imaging technologies and genomic innovations has significantly impacted the landscape of medical research and clinical practice, particularly in oncology. This synthesis will delve into the advances in imaging technologies, genomic innovations, and the implications for clinical trials and translational research.

### 9.1. Advances in Imaging Technologies

High-resolution imaging techniques have revolutionized the ability to visualize and characterize tumors with unprecedented clarity. Techniques like high-field magnetic resonance imaging (MRI) and advanced computed tomography (CT) have enhanced the detection of smaller lesions and the evaluation of tumor microenvironments [123,124]. These advancements allow for more accurate staging of cancer, which is crucial for determining appropriate treatment strategies. For instance, high-resolution imaging can reveal subtle changes in tumor morphology and vascularity, providing insights into tumor aggressiveness and potential treatment responses. Furthermore, the integration of functional imaging techniques, such as diffusion-weighted MRI and dynamic contrast-enhanced MRI, enhances the ability to evaluate tumor perfusion and cellularity, further informing clinical decision-making [124,125].

### 9.2. Genomic Innovations

Single-cell sequencing technologies have emerged as powerful tools for dissecting the heterogeneity of tumors at an unprecedented resolution. By analyzing individual cells, researchers can uncover the diverse genetic and epigenetic landscapes within a tumor, revealing insights into tumor evolution, metastasis, and treatment resistance [126,127]. This level of detail is crucial for understanding the mechanisms underlying cancer progression and for identifying potential therapeutic targets. For instance, single-cell RNA sequencing has been utilized to characterize the tumor microenvironment and identify distinct cellular subpopulations that may contribute to therapeutic resistance [128]. The insights derived from single-cell sequencing can significantly inform the development of targeted therapies tailored to the specific characteristics of an individual patient’s tumor.

Combining different types of biological data, such as genetics, gene activity, proteins, and metabolites, helps us understand cancer better. This approach reveals how these biological layers interact and influence tumor behavior [126]. For example, integrating genomic data with proteomic profiles can reveal how specific mutations influence protein expression and function, thereby affecting treatment responses [129,130]. Studies have demonstrated that the integration of genomic and proteomic data has been successfully applied in the context of non-small cell lung cancer (NSCLC), where mutations in the *EGFR* gene have been shown to correlate with specific protein expression patterns that predict responsiveness to EGFR tyrosine kinase inhibitors [131]. Similarly, in breast cancer, *HER2* amplification has been linked to the expression of HER2 proteins, guiding the use of *HER2*-targeted therapies [132]. The application of multi-omics data in clinical settings holds promise for developing personalized treatment strategies that consider the unique molecular profile of each patient’s tumor, ultimately improving the outcomes [127].

Recent advancements in multi-omics integration methodologies, such as those proposed by Yin et al., have highlighted the potential of using robust graph neural networks to classify cancer subtypes based on integrated multi-omics data, further supporting the clinical relevance of this approach [133]. Additionally, Nicora et al. emphasized the utility of machine learning methods in analyzing multi-omics data, which can facilitate the identification of novel therapeutic targets and improve treatment stratification in complex diseases like pancreatic adenocarcinoma [134]. Moreover, the work of Wang et al. [135] illustrates how multi-omics integration can lead to biomarker identification and patient classification, particularly in breast cancer, thereby enhancing the precision of treatment strategies.

The integration of multi-omics data not only aids in understanding the molecular mechanisms underlying cancer but also enhances the ability to predict treatment responses. For example, Liu’s research on DNA methylation-based classification models demonstrates how combining genomic and epigenomic data can improve cancer prognostication [118]. As the field continues to evolve, the incorporation of multi-omics approaches into clinical practice is expected to yield significant improvements in patient outcomes by tailoring therapies to the specific molecular characteristics of individual tumors.

### 9.3. Clinical Trials and Translational Research

Numerous ongoing clinical trials are exploring the application of advanced imaging techniques and genomic innovations in cancer treatment. These studies aim to validate the utility of radiogenomic signatures in predicting treatment responses and outcomes, thereby enhancing personalized medicine approaches [55]. Future trials are expected to focus on integrating AI-driven imaging analysis with genomic data to refine patient stratification and optimize therapeutic interventions. For instance, trials investigating the efficacy of combination therapies based on specific radiogenomic profiles are underway, intending to overcome resistance mechanisms in breast cancer and other malignancies [135].

### 9.4. Incorporation of AI in Radiogenomics

The integration of artificial intelligence (AI) into imaging technologies and genomic innovations is poised to significantly enhance the accuracy and efficiency of cancer diagnosis and treatment. AI technologies, particularly deep learning algorithms, have shown promise in analyzing complex imaging data and correlating them with genomic information, thereby facilitating the development of personalized treatment strategies. AI-driven imaging analysis has transformed traditional methods of tumor detection and characterization. For instance, convolutional neural networks (CNNs) have been employed to identify radiogenomic associations in breast cancer, demonstrating superior performance compared to conventional image analysis techniques [136]. These algorithms can process vast datasets, extracting intricate patterns that may elude human observers, thus improving diagnostic accuracy and reducing the interpretation time [131]. Moreover, AI has been instrumental in predicting genetic mutations from imaging data, as evidenced by studies that achieved high accuracy in identifying isocitrate dehydrogenase (IDH) mutations in gliomas using deep learning approaches [137]. This capability not only enhances the diagnostic precision but also aids in stratifying patients for targeted therapies based on their unique tumor profiles.

The application of AI in radiogenomics also addresses some limitations faced by clinicians in utilizing imaging data effectively. Traditional methods often rely on a subjective interpretation, which can lead to variability in the diagnosis and treatment planning. AI algorithms, in contrast, provide a more objective and reproducible assessment of imaging data, thereby minimizing human error [138]. However, the rapid integration of AI into clinical practice raises concerns regarding the need for robust validation of these technologies. A systematic review highlighted the importance of rigorous design and reporting standards for AI studies to ensure their clinical applicability and safety [139]. Furthermore, interdisciplinary collaboration between engineers and healthcare professionals is essential to develop AI tools that meet the actual requirements of personalized medicine. The incorporation of AI into imaging and genomic analyses represents a significant advancement in oncology with the potential to enhance diagnostic accuracy, optimize treatment strategies, and ultimately improve patient outcomes. As ongoing research continues to refine these technologies, the future of cancer management will likely be characterized by increasingly personalized approaches that leverage the power of AI to integrate complex biological data.

The translation of radiogenomic research into clinical practice is critical for improving patient care. Efforts are being made to establish standardized protocols for the integration of imaging and genomic data in routine clinical workflows [57]. These include the development of decision-support tools that leverage AI algorithms to assist clinicians in interpreting complex radiogenomic data and making informed treatment decisions. Additionally, educational initiatives aimed at enhancing genomic literacy among healthcare professionals are essential for fostering the adoption of these innovative approaches in clinical settings. By bridging the gap between research and practice, the potential of radiogenomics to revolutionize cancer management can be fully realized. The advances in imaging technologies and genomic innovations are highlighted by reshaping the landscape of cancer research and treatment. The integration of high-resolution imaging, AI-driven analysis, single-cell sequencing, and multi-omics data holds great promise for enhancing personalized treatment strategies, improving predictive and prognostic capabilities, and facilitating the translation of research findings into clinical practice.

## 10. Limitations

Despite the promising potential of radiogenomics in addressing resistance mechanisms in metastatic endocrine-positive breast cancer, several limitations must be acknowledged. One of the primary challenges is the limited availability of comprehensive patient data, which restricts the robustness and generalizability of radiogenomic findings. Many studies rely on small patient cohorts, leading to biased results and hindering the validation of identified biomarkers across diverse populations [67,140]. To overcome this limitation, expanding patient databases with a diverse demographic representation is essential, ensuring the development of universally applicable radiogenomic tools [66,141,142]. Another significant challenge lies in the variability of imaging protocols across different institutions and studies. Differences in imaging modalities, acquisition parameters, and interpretation methods can introduce inconsistencies, complicating the standardization of radiogenomic findings [57,143]. This variability complicates efforts to compare and integrate data across studies, which is crucial for establishing reproducible and clinically relevant biomarkers [144]. Furthermore, concerns regarding the reproducibility of radiogenomic analyses stem from the reliance on advanced computational methods, which often lack standardized workflows and are susceptible to variations in preprocessing and algorithm selection. Addressing these challenges will require the development and adoption of standardized imaging protocols and computational pipelines to enhance the reproducibility and clinical utility of radiogenomic research [141,145]. In addition to these challenges, the integration of radiogenomics into clinical practice faces obstacles related to the interpretation of complex data. The need for interdisciplinary collaboration among radiologists, oncologists, and bioinformaticians is paramount to effectively translate radiogenomic findings into actionable clinical insights [146]. Moreover, the evolving nature of imaging technologies and genomic analyses necessitates continuous education and training for healthcare professionals to keep pace with advancements in the field [147,148]. By addressing these multifaceted challenges, the field of radiogenomics can move closer to realizing its full potential in improving the outcomes of patients with metastatic endocrine-positive breast cancer.

## 11. Conclusions

The radiogenomic landscape of metastatic endocrine-positive breast cancer resistant to aromatase inhibitors is a complex interplay of genetic alterations and treatment responses that significantly impact patient outcomes. Understanding the mechanisms of resistance to aromatase inhibitors is crucial, as this knowledge can inform therapeutic strategies and improve clinical management. Resistance mechanisms in endocrine-positive breast cancer are multifaceted, involving genomic alterations such as mutations in *ESR1* and other signaling pathways, including the MAPK and PI3K pathways, which have been shown to contribute to treatment failure [71,149,150]. For instance, *ESR1* mutations are prevalent in patients who develop resistance to aromatase inhibitors, serving as a prognostic marker for poor outcomes [151]. Furthermore, identifying additional mutations, such as those in *NF1* and *ERBB2*, highlights the genetic diversity and adaptability of metastatic tumors under selective pressure from endocrine therapies [152,153]. The role of radiogenomics in this context cannot be overstated. Radiogenomics is an innovative approach that merges advanced imaging data with comprehensive genomic information to enhance our understanding of tumor biology and the tumor response to various treatments. By analyzing these two types of data together, researchers and clinicians can uncover specific biomarkers that are associated with resistance to therapies. This knowledge empowers healthcare professionals to tailor treatment plans to meet the unique needs of each patient, ultimately improving the effectiveness of cancer care and increasing the chances of positive outcomes [79,147,154]. For example, the use of a circulating tumor DNA (ctDNA) analysis can reveal clonal evolution and the emergence of resistant subclones, which is critical for understanding treatment dynamics and guiding subsequent therapeutic interventions [155]. Moreover, advancements in imaging techniques can help visualize tumor heterogeneity and monitor treatment responses in real time, further enhancing personalized treatment strategies. The implications for future clinical practice are profound. The potential for personalized medicine in the treatment of metastatic endocrine-positive breast cancer is becoming increasingly feasible. By leveraging the insights gained from radiogenomics, clinicians can develop more effective treatment regimens that account for the unique genetic makeup of each patient’s tumor. This could involve the combination of aromatase inhibitors with CDK4/6 inhibitors, which have shown promise in overcoming resistance and improving progression-free survival [79,155,156]. Additionally, integrating novel therapeutic agents targeting specific mutations, such as those in *ESR1* or *RET*, may further enhance treatment efficacy and patient outcomes [157]. The radiogenomic landscape of metastatic endocrine-positive breast cancer resistant to aromatase inhibitors poses both challenges and opportunities. A deeper comprehension of resistance mechanisms combined with the innovative application of radiogenomics are crucial for advancing personalized medicine in this field. Concentrating on integrative approaches that merge genomic insights with clinical practice can enhance patient outcomes and potentially revolutionize the treatment landscape for this complex cancer subtype.

## Figures and Tables

**Table 1:** Key biomarkers and their applications in imaging techniques.

Biomarker	Aging or Tumor Grading	MRI Application	CT Application	PET Application	References
Ki-67	Tumor grading	Identifies proliferative activity in tumor regions	Limited application	Assesses metabolic activity via radiolabeled tracers	[2]
*p53*	Tumor grading	Evaluates alterations linked to tumor aggressiveness	Identifies structural abnormalities	Highlights p53-related metabolic dysregulation	[3]
VEGF	Tumor grading	Monitors angiogenesis within tumor microenvironments	Detects neovascularization	Tracks VEGF expression in angiogenic processes	[51]
Telomere Length	Aging	Assesses tissue-specific telomere attrition	Limited application	Tracks telomere-associated cellular senescence	[2,6]
*HER2*	Tumor grading	Identifies HER2 overexpression in breast tumors	Detects anatomical changes in HER2-positive tumors	Assesses HER2-positive metabolic pathways	[64]
GLUT1	Tumor grading	Highlights hypoxic regions with altered glucose uptake	Detects metabolic reprogramming	Quantifies glucose metabolism with FDG radiotracers	[51]
Lamin A/C	Aging	Evaluates nuclear structure changes related to aging	Limited application	Tracks the senescence-associated nuclear architecture	[2,6]
MMPs	Tumor grading	Identifies extracellular matrix remodeling	Detects invasion-related structural changes	Monitors tumor invasion and metastasis dynamics	[2,6]

## Data Availability

The data were obtained from a computer-assisted search of PubMed and Scopus.
